# Enhancing patient safety through radiograph rejection analysis: Insights from a diagnostic imaging department audit

**DOI:** 10.12669/pjms.41.7.11441

**Published:** 2025-07

**Authors:** Muhammad Yaseen, Tahira Nishtar, Rahaf Ajaj, Amir Ali

**Affiliations:** 1 Muhammad Yaseen, MPhil. Department of Radiology, Lady Reading Hospital (LRH), Peshawar, Pakistan; 2 Tahira Nishtar, FCPS (Radiology). Department of Radiology, Lady Reading Hospital (LRH), Peshawar, Pakistan; 3 Rahaf Ajaj, Ph.D. Environmental Health & Safety Department, Abu Dhabi University, UAE; 4 Amir Ali, MPhil. Department of Radiology, Lady Reading Hospital (LRH), Peshawar, Pakistan

**Keywords:** Artifact, Labeling, Rejection analysis, Quality assurance, positioning errors

## Abstract

**Objective:**

Assessment of actual radiograph rejection was the primary goal of this investigation in the imaging facility of a public sector hospital, which was noticeably high during the pandemic as staff members were afraid of infection.

**Methods:**

This retrospective study was carried out in the department of Radiology, LRH from July 2023 to March 2024 during which a total of 47,300 radiographic examinations were conducted. Analysis was performed for the rejected radiographs and their causes respectively.

**Results:**

Our rate of 11% is comparable to the global range of 3.4-14.1 % respectively. Of the total rejection, 46.1% rejection was observed in males and 53.9% in females while 69.5 % & 30.5 % rejection was noticed in adults and pediatric age group respectively. Artifacts (26.2%) was the prime cause of rejection followed by motion (18.8%), positioning (14%), improper collimation (11.7%), exposure errors (8.4 %), wrong labeling (6.6%), machine faults (5.7 %), detector errors (4.5%), PACS issues (2.5 %) and re-requests from referring physician (1.6%). The highest rejection with respect to anatomical body parts was observed in chest radiography (29.9%), followed by extremities (28.8%), spine (11.4%), KUB (9.9%), skull (8.4%), abdomen (5.5%), pelvis (4.4%), and neck (1.7%) respectively.

**Conclusion:**

Radiograph rejection has reduced considerably but still is a common problem within the facility due to many contributing factors. Implementation of rejection analysis as an integral part of the quality assurance program as well as focusing on staff-centered skill and knowledge upliftment training programs can result in a significant reduction enhancing patient safety.

## INTRODUCTION

Diagnostic imaging using x-rays represents the common examinations in medicine, account for the most remarkable manmade source of radiation exposure to the public.^1^ During radiological procedures, both patients and staff are exposed to radiations. Therefore, the judicious use of radiation is more beneficial and safe if the benefits of radiation are higher than the associated radiation risks.

In diagnostic imaging, one of the main goals of quality assurance (QA) program is to produce consistently high-quality radiographs with minimum exposure to patient.^2^,^3^ The objective of radiological examination should be to create images of sufficient quality for the clinical task, not of unnecessarily high quality that raises the radiation dose.^4^,^5^ The risk of malignancy development in later stages increases if a patient undergoes repeated x-ray examinations as reported.^6^

However, using too low doses can also be detrimental, if it results in images that cause a clinical error leading to repetition.^7^ Thus, rejection analysis is an integral part of the primary QA program and is a well-established practice in diagnostic imaging to evaluate image quality.^8^ Implementation of radiation safety principles can be very fruitful in minimizing the harmful effects of ionizing radiations, and this can be achieved by addressing department specific issues and through well-trained staff with adequate radiographic techniques and a strong grip over radiation safety protocols.^9^ Poor image quality, improper collimation, artifacts, motion, and equipment malfunction tends to increase the rejection rate causing patients repeated examinations.^10^ These repeated radiographs increases patient doses, decrease equipment lifespan, increase operational costs with longer waiting times, ultimately undermining departmental efficiency along with higher patient dissatisfaction.^11^-^13^

Radiograph rejection in digital radiography is less common provided wide exposure latitude as compared to screen-film and computed radiography with narrow exposure latitude and increased manual interventions resulting in frequent rejections contributing to unnecessary patient radiation doses.^14^ Post-image processing and manipulation with wide range of exposure in digital systems, have more room of adjusting image quality as compared to conventional systems.^15^ However, with the evolution of panel technologies and ease of acquisitions, rejection is still a problem in digital radiography.

Medical imaging has evolved drastically with DR systems as part of standard patient care with smooth service delivery.^16^ In digital systems, artifacts or positioning errors results in rejection.^17^ Assessment of the quantity and causes of rejection can be best performed by making reject analysis program (RAP) as an essential part of the QA program.^18^ This will not only help in producing good quality images but also aid in reduction of patient radiation doses, lowering extra costs in repeating examinations along with prolonging the tube life.

Radiograph rejection of our facility was high during the COVID-19 pandemic^19^ because of the fear of infection from coronavirus disease. Thus, the radiographers even with necessary precautionary clothing’s against infection were afraid to touch and position patient as per set standards from close proximity. Therefore, radiographers used verbal commands for positioning patients resulting in high rejections as patients were unaware of proper body part orientation. Thus, basic aim of this study was to assess the actual rejection rate, which was considerably high during the coronavirus period, to find out causes of rejection and to identify the weak zones where further improvement can be made towards achieving good quality images with minimal patient radiation doses along with skill enhancement for our staff.

## METHODS

This retrospective study was conducted at Radiology Department- LRH, Peshawar from July 2023 to March 2024 [Non-COVID phase] and a total of 47,300 radiographic examinations were carried out on four digital X-ray systems.

In addition to written request, all radiographs were produced upon online requests by the referring physician. Radiation exposure was justified with all required protection measures before any radiograph was taken. The formula used to calculate the rejection rate was:


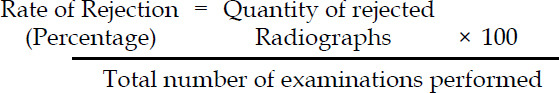


### Statistical analysis:

In this study, a supervisor and senior radiographer were responsible for rejection; therefore level of agreement and degree of variation as observers was determined. Kappa Statistics were used on a sample of 200 images segregated into 25 images per anatomical part included in the study and substantial agreement (0.61-0.80) was found between them. The radiographer was primarily responsible for the rejection which was endorsed by both. Detailed statistical analysis was not required for this study. After entering the data into Microsoft Excel, the findings were analyzed and presented in the form of graphs and percentages.

### Ethical approval:

It was obtained from the Institutional Review Board (IRB) under Reference No: 349/LRH/MTI; dated September 19, 2024.

### Rejection criteria:

The majority of the rejection was performed by an expert radiographer with relevant experience of more than 15 years under supervision of imaging supervisor having over 20 years of experience. Finally, images were viewed from PACS and analyzed. Variability and agreement between the observers was checked using Kappa statistics showing good agreement (0.61-0.80). Since, the radiographer determines whether or not the radiographs, should be rejected. However, occasionally, when the radiographer was unsure whether or not to reject an image, the supervisor made the final decision. According to the report of Task Group 151 of the AAPM Imaging Physics Committee, 10 standardized causes of rejection were used to aggregate the number and reasons for rejection in dedicated proforma.^2^

## RESULTS

**Fig.1:**
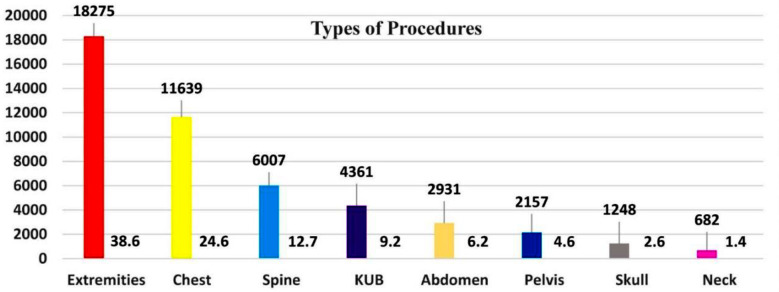
Distribution of procedures performed as per anatomical body parts.

**Fig.2:**
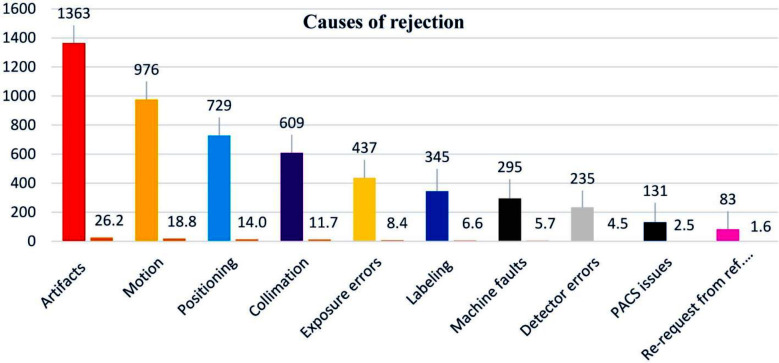
Rejected radiographs with respect to causes of rejection.

**Fig.3:**
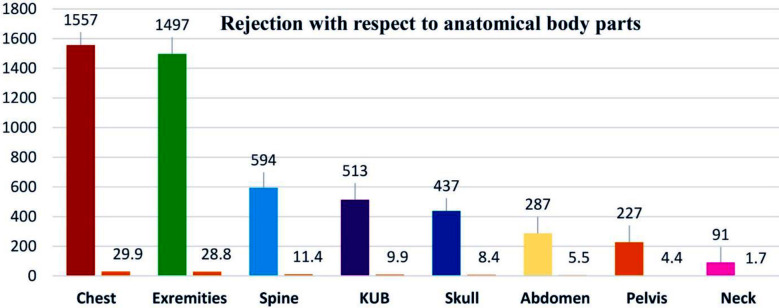
Rejected radiographs as per anatomical body part.

Out of the total 47,300 imaging examinations were carried out from July 2023 to March 2024, the gender distribution shows 24,921 (52.7%) males and 22,379 (47.3%) female patients, while 38,754 (81.9%) were adults and 8,546 (18.1%) patients were of pediatric age group. Similarly, in accordance with body parts, the distribution of procedures was: Extremities: 18,275 (38.6%), including both upper and lower with subparts: chest: 11,639 (24.6%), spine: 6,007 (12.7%), KUB: 4,361 (9.2%), abdomen: 2,931 (6.2%), pelvis: 2,157 (4.6%), skull: 1,248 (2.6%) and neck: 682 (1.4%), as illustrated in Fig.1. A total of 5,203 radiographs were rejected during the study period. 2,394 (46.1%) and 2,809 (53.9%) radiographs were rejected in males and females respectively while 3,616 (69.5%) were rejected in adults and 1,587 (30.59%) in pediatrics. Artifacts (26.2%) was the most common cause of rejection followed by motion 18.8%, positioning 14%, improper collimation 11.7%, exposure errors 8.4%, wrong labeling 6.6%, machine faults 5.7%, detector errors 4.5%, PACS issues 2.5% and referring physician re-requests 1.6% respectively as shown in Fig2. The highest rejection was observed in chest radiography (29.9%), followed by extremities (28.8%), spine (11.4 %), KUB (9.9%), skull (8.4%), abdomen (5.5%), pelvis (4.4%), and neck (1.7%), respectively, as shown in Fig.3.

## DISCUSSION

This study highlighted the actual rejection rate of the facility which was 11% which is lower than the rejection rate during the COVID period [Aug- Nov- 2020] which was 17% mainly due to the fear of infection leading to positioning errors as technical staff were reluctant to touch patients during positioning since patients were not conversant with self-positioning based on verbal communication.^19^ The current rejection has dropped substantially but still higher with digital radiography system which needs further reduction to ensure safety of patients from being exposed to repeated radiation doses.^20^

Rejection analysis is an important part of the quality assurance (QA) program in radiology department providing radiography services to ensure reduction in the rejection factors so that expenses, patient flow and radiation doses to patients and staff can be reduced.^21^ This practice not only provides fruitful information regarding institutional rejection rate but, importantly, it highlights basic causes behind the rejection to counter accordingly in order to improve the quality of images, reduce number of rejections, minimize patient exposures, decrease costs, optimize machine performance and life expectancy and lower staff burden, contributing to smooth patient service delivery with optimum image quality.^22^-^24^

The rejection rate observed in this current study was 11% which is much lower than the findings of our previous study conducted during the COVID-19 pandemics showing 17%. This was mainly due to the fear of infection leading to positioning errors as technical staff were reluctant to touch patients during positioning since patients were not conversant with self-positioning based on verbal communication which was also reported in another study.^25^ The current rejection reflects the actual rejection of the facility, which is consistent with relevant studies reporting 14.1% in Ghana^8^, 11%-12% in Norway^11^, 3.6% in Taiwan^26^ and 11.15% in Iran.^27^ The rejection rate has reduced considerably as compared to previous findings but is still high which can be further reduced by properly implementing patient preparation protocols, ensuring streamlined communication and by enhancing technical skills of staff through continuous professional training.

The basic causes of rejection were artifacts (26.1 %) which was also followed by motion (18.8%) while positioning was third largest cause of rejection (14%) and others were less prominent, as shown Fig.2. Artifacts contributed more rejections in females as jewelry is extensively used as part of tradition and culture and are reluctant to remove it during positioning. Secondly, hair pins, clips and other female accessories, along with pockets accessories of males were the contributing factors in rejection reflecting inadequate implementation of patient preparation standards, improper education regarding their procedures, illiteracy and high patient influx resulting in discomfort among the radiographers. The frequent use of immobilization gadgets, especially for uncooperative traumatic patients, elderly and pediatric patients who rarely hold their orientation resulted in rejection due to unwanted motion. Positioning errors reduced considerably in present study as staff came out of infection phobia implementing positioning protocols properly as highlighted in (Table-I).

**Table-I:** Comparison of rejected radiographs with relevant studies w.r.t. anatomical body parts [Non-COVID phase].

Body Parts	Current Study		Previous study^19^		Alyousef, et al.^10^		Weatherburn, et al.^18^	
	n	% (N=5203)	n	% (N=2550)	n	% (N=455)	n	% (N=525)
Extremities	1497	28.8	1125	44.1	196	43	105	20
Chest	1557	29.9	593	23.3	141	31	235	44.8
Spine	594	11.4	290	11.4	36	8	88	16.8
Abdomen	287	5.5	162	6.4	41	9	40	7.6
Skull	437	8.4	150	5.9	18	4	47	8.9
Pelvis	2275	4.4	120	4.7	23	5	10	1.9
KUB	513	9.9	95	3.7				
Neck	91	1.7	15	0.6	-	-	-	-

It was surprising that chest radiography represented the highest rejection rate of 29.9% despite the fact that extremities were the most frequently performed procedures (38.6%) among all types. This high rejection rate was largely due to artifacts, which appeared in females because of non-compliance with patient preparation protocols, motion during positioning in older adults and pediatric patients, and breath-holding issues in adults. Extremities reported the second highest number of rejections (28.8%), possibly due to positioning errors and improper collimation. Spine procedures also showed an 11.4% rejection rate due to exposure issues, artifacts from pocket items in males, and motion. The rejection rate for KUB procedures was 9.9%, primarily due to improper utilization of patient positioning guidelines, artifacts, and poor area of interest coverage.

**Fig.4:**
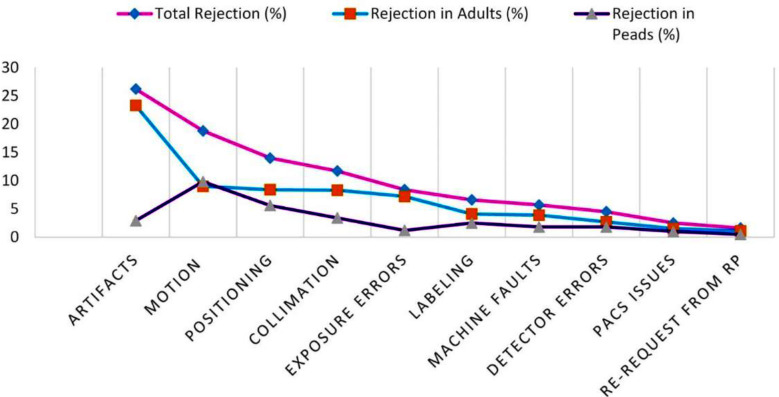
Comparison of rejection in adults and pediatric age group with respect to causes of Rejection.

**Fig.5:**
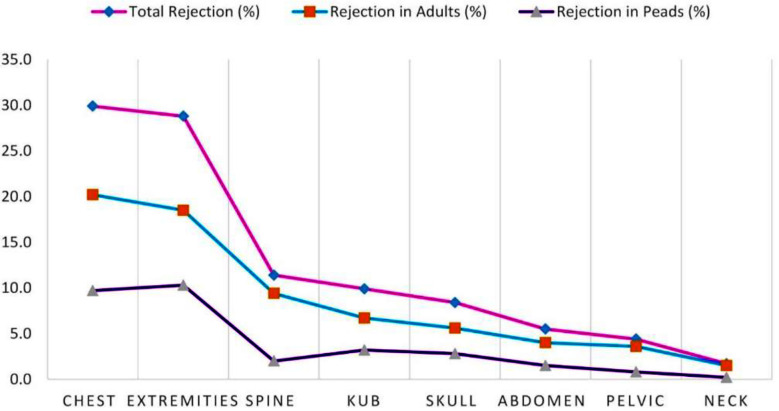
Comparison of rejection in adults and pediatric age group with respect to anatomical body parts.

This was followed by skull radiography, with 8.4% rejection due to positioning uncertainties and inaccurate projection angle settings. Rejection rate for other body parts were less prominent and consistent with published data from Table-II. Gender group’s comparison of rejected radiographs revealed that rejection in females was high (53.9%) while it was 46.1% in males because of the appearance of artifacts which were more common in females than males due to extended use of jewelry and other accessories which mostly appeared on images due to non-compliance of patient preparation protocols. Rejection in adults was higher (69.5%) compared to the pediatrics (30.5%) because the number of procedures performed in adults was far higher (81.9%) compared to the pediatric age group (18.1%). It was also observed that positioning errors, improper collimation, anxiety and artifacts are more common in adults. However, motion contributed to more rejection in pediatric age group as they are more non-cooperative as shown in Fig.4. While with respect to body parts, chest radiography showed highest rejection, followed by extremities and others in comparison between adults and the pediatric age group, as evident from Fig.5 respectively.

**Table-II:** Comparison of rejected radiographs with relevant studies in terms of their causes [Non-COVID phase].

Causes of Rejection	Current Study		Previous Study^19^		Alyousef, et al.^10^		Chih-Sheng, et al.^26^	
	n	% (N=5203)	n	% (N=2550)	n	% (N=455)	n	% (N=4812)
Artifact	1363	26.2	573	22.4	59	13	990	20.6
Motion	976	18.8	310	12.16	-	-	577	11.99
Positioning	729	14	777	30.5	64	14	2697	56.1
Exposure Error	437	8.4	175	6.9	27	6	193	4

**Fig.6:**
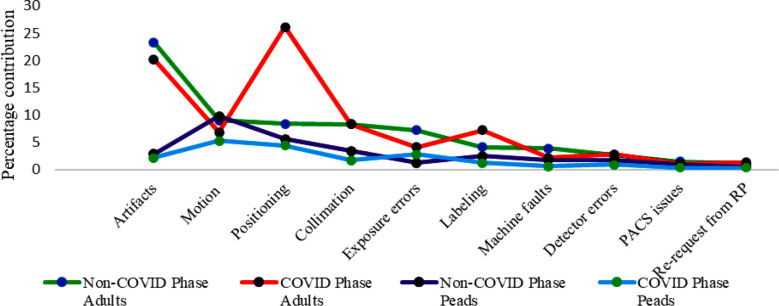
Comparative analysis of causes of rejection in adults and Peads in COVID Phase versus Non-COVID phase.

Analysis of rejection is an important quality control parameter in radiography and is used to identify the basic reasons for rejection which can be addressed accordingly to reduce patient and staff radiation doses.^28^-^30^ Therefore, rejection analysis should be performed once on quarterly basis as per the recommendations of AAPM.^2^ The study results reflect clear insights into the rejection rate of the facility and its causes, revealing poor execution of patient positioning protocols. This might be due to the high patient influx causing discomfort among staff, poor cooperation from patients, lack of proper communication and other contributing factors. The comparison of causes of rejection in the COVID phase versus Non-COVID Phase between adults and pediatric patients highlighted that positioning was the key contributor in rejection in the former phase (COVID phase) due to fear of infection among technical staff in going close to patient during positioning. While in normal working circumstances (Non-COVID phase), the contribution of positioning in rejection was not significant as compared to other causes i.e. artifacts, motions etc. as illustrated in Fig.6.

These shortcomings can be well addressed by taking the inputs of technical staff in streamlining patient workflow along with other specific needs by conducting regular trainings modules on patient positioning, implementation of protocols along with dose optimization techniques to enhance the skills and expertise of staff which will increase their expertise resulting in less rejection as reported in several studies^31^-^33^, setting new protocols along with workflow adjustments to further reduce rejection rate and keeping pace with new technological advancements and developments in modern era of imaging to achieve optimum image quality with enhanced patient safety.

### Strengths of the study:

This study explored the various aspects of x-ray rejection in imaging facilities and gave insight details of the causes of rejection and the technical gaps of staff which needs to be addressed frequently to improve image quality with less rejection. Addressing these issues promptly can enhance image quality, reduce rejections, improve service delivery, ensure patient safety, and boost staff skills.

### Limitations:

This study focused only on X-ray procedures performed in the main imaging department during study period, excluding those conducted in the emergency department and portable cases in wards. A broader analysis across all imaging modalities is needed for a comprehensive strategy to enhance image quality and minimize radiation exposure along estimation of doses resulting from these repeated procedures along with expenses incurred as a result of the rejected radiographs.

## CONCLUSION

The study identified the actual rejection rate of the facility which is considerably high with digital systems. Substantial drop in rejection can be achieved by introduction of quarterly audits of rejected radiograph along with integration of automatic rejection software tracking system with picture archiving and communication system (PACS) which will be helpful in identification of causes for rejection, staff requisites and highlighting their technical needs through regular monthly staff-focused skill upliftment sessions and streamlining workflow adjustments resulting in improved image quality enhancing patient safety as well.
